# Use of Different Anti-PD-1 Checkpoint Combination Strategies for First-Line Advanced NSCLC Treatment—The Experience of Ion Chiricuță Oncology Institute

**DOI:** 10.3390/cancers16112022

**Published:** 2024-05-26

**Authors:** Alexandra-Cristina Preda, Tudor-Eliade Ciuleanu, Nicolae Todor, Cătălin Vlad, Dana Ioana Iancu, Cristina Mocan, Mariana Bandi-Vasilica, Florina Albu, Irina Mihaela Todor-Bondei, Mădălina Claudia Hapca, Milan-Paul Kubelac, Adelina Dadiana Kubelac-Varro

**Affiliations:** 1 Oncology Institute Prof. Dr. Ion Chiricuță, 34–36 Republicii Street, 400015 Cluj-Napoca, Romania; preda.alexandra_cristina@yahoo.com (A.-C.P.); tudor_ciuleanu@hotmail.com (T.-E.C.); tdrnicolae@gmail.com (N.T.); catalinvlad@yahoo.it (C.V.); dana_iancu2004@yahoo.com (D.I.I.); mocan.cristina@gmail.com (C.M.); bandi.vmariana@gmail.com (M.B.-V.); florinaalbu18@gmail.com (F.A.); airina202003@yahoo.com (I.M.T.-B.); 2 Iuliu Hațieganu University of Medicine and Pharmacy, 8 Victor Babeș Street, 400012 Cluj-Napoca, Romania; madalina.prodan06@gmail.com (M.C.H.); dadi.varro@gmail.com (A.D.K.-V.)

**Keywords:** NSCLC, immunotherapy, prognostic factors

## Abstract

**Simple Summary:**

Different immunotherapy combinations improved prognosis for advanced non-oncogene driven NSCLC, but they were not directly compared. The aim of our study is to present the real-world data for 122 patients treated at the Institute of Oncology in Cluj-Napoca with three different consecutive immunotherapy combinations (dual immunotherapy—18 patients, dual immunotherapy plus short course chemotherapy—33 patients, mono-immunotherapy plus full course chemotherapy—71 patients). Efficacy results using different immunotherapy combination strategies were in line with those from the registration trials, with 22.2 months median overall survival and 49% actuarial 2-year survival. Results were not significantly different between the three protocols. Older age, impaired performance status, corticotherapy in the first month of immunotherapy, and >3.81 neutrophils to lymphocytes ratio were independent unfavorable prognostic factors in the multivariate survival analysis. Long-term data are available for the dual immunotherapy cohorts, with 30.5% and 18.8% of patients alive at 5 years.

**Abstract:**

Purpose. Different combination modalities between an anti-PD-1/PD-L1 agent and a platinum-based chemotherapy or another checkpoint inhibitor (with or without a short course or full course of a platinum doublet) proved superior to chemotherapy alone in multiple clinical trials, but these strategies were not directly compared. The aim of this study is to report the real-world data results with different immunotherapy combinations in a series of patients treated in consecutive cohorts at the Ion Chiricuță Oncology Institute. Methods. A total of 122 patients were successively enrolled in three cohorts: (1A) nivolumab + ipilimumab (18 patients), (1B) nivolumab + ipilimumab + short-course chemotherapy (33 patients), and (2) pembrolizumab plus full-course chemotherapy (71 patients). Endpoints included overall survival (OS), progression-free survival (PFS), objective response (ORR), and univariate and multivariate exploratory analysis of prognostic factors. RESULTS. Median follow-up in the consecutive cohorts 1A, 1B, and 2 was 83 versus 59 versus 14.2 months. Median OS and PFS for all patients were 22.2 and 11.5 months, respectively, and 2-year actuarial OS and PFS were 49% and 35%, respectively. For the nivolumab + ipilimumab (cohorts 1A and 1B) versus pembrolizumab combinations (cohort 2), median OS was 14 vs. 24.8 months (*p* = 0.18) and 2-year actuarial survival 42% vs. 53%; median PFS was 8.6 vs. 12.7 months (*p* = 0.41) and 2-year actuarial PFS 34% vs. 35%; response rates were 33.3% vs. 47.9% (*p* = 0.22). Older age, impaired PS (2 versus 0–1), corticotherapy in the first month of immunotherapy, and >3.81 neutrophils to lymphocytes ratio were independent unfavorable prognostic factors in the multivariate analysis of survival (limited to 2 years follow-up). The 5-year long-term survival was 30.5% and 18.8% for cohorts 1A and 1B, respectively (not enough follow-up for cohort 2). Conclusions. Efficacy results using different immunotherapy combination strategies were promising and not significantly different between protocols at 2 years. Real-world efficacy and long-term results in our series were in line with those reported in the corresponding registration trials.

## 1. Introduction

Use of checkpoint inhibitors targeting PD-1 or PD-L1 either alone or in different combinations with standard platinum-based chemotherapy or with another checkpoint inhibitor (with or without chemotherapy) has shown improved survival over standard platinum-based chemotherapy as frontline treatment in advanced non-oncogene driven NSCLC [1,2,3,4,5,6,7,8,9,10,11,12]. Mono-immunotherapy (with pembrolizumab—KeyNote-024 [9] and KeyNote-042 [6], atezolizumab—IMpower-110 [4], or cemiplimab—EMPOWER Lung 1 [10]) is recommended for patients highly expressing PD-L1, while combination strategies seem efficacious irrespective of the PD-L1 status.

The experimental arms of the clinical trials with pembrolizumab + platinum + pemetrexed (KeyNote-189) [1], atezolizumab + carboplatin + paclitaxel + bevacizumab (IMpower-150) [11], and atezolizumab + carboplatin + nab-paclitaxel (IMpower-130) [12] for non-squamous NSCLC, pembrolizumab + platinum + paclitaxel for squamous NSCLC (KeyNote-407) [8], and cemiplimab + platinum doublet (EMPOWER Lung 3) [2] for both squamous and non-squamous NSCLC, all became recommended standard treatments, irrespective of the PD-L1 status.

On the other hand, other clinical trials combined two checkpoint inhibitors (nivolumab + ipilimumab—CheckMate-227) [3] or added to the dual immunotherapy combination a short course of chemotherapy (nivolumab + ipilimumab + two cycles of a platinum doublet—CheckMate-9LA) [7] or a complete course of chemotherapy (durvalumab + tremelimumab + four cycles of chemotherapy +/− maintenance in non-squamous NSCLC—Poseidon trial) [5]. Dual nivolumab + ipilimumab immunotherapy is authorized in the US in PD-L1 positive patients, and the other combinations are authorized in the US and Europe irrespective of the PD-L1 status.

The present study aimed to analyze the real-world data results obtained in a single institution (Prof. Dr. Ion Chiricuță Institute of Oncology, Cluj-Napoca, Romania) using anti-PD-1 immunotherapy in different combinations.

The association strategies included three cohorts: cohort 1A, dual immunotherapy with nivolumab + ipilimumab, like in CheckMate-227; cohort 1B, dual immunotherapy with nivolumab + ipilimumab + short-course platinum-based chemotherapy (like in CheckMate-9LA); cohort 2, pembrolizumab + four cycles of standard platinum-based chemotherapy followed by maintenance for non-squamous NSCLC histology (like in KeyNote-189 and KeyNote-407). These strategies were used in different time periods, as the different checkpoint inhibitors became available for use in Romania, so data were obtained from successive cohorts and the study was non-randomized. We intended to do a real-world institutional study, including all the experience gained with the different checkpoint inhibitors’ combinations, mentioning the different follow-up times for each successive cohort. A comparison between the three different treatments (cohorts 1A, 1B, and 2) or the two different strategies (cohorts 1A and 1B vs. 2) and the analysis of prognostic factors was possible only at 2 years, due to a shorter follow-up in cohort 2. On the other hand, for cohorts 1A and 1B, a long-term survival analysis was possible and separately conducted, due to the long follow-up time with these treatments.

## 2. Patients and Methods

This study included adult patients who were diagnosed with advanced non-small cell lung carcinoma (stage IV or recurrent cancer cases restaged according to AJCC 8th edition guidelines). These patients had histopathological confirmation, did not have EGFR or ALK activating mutations, had not received any previous systemic therapy for advanced disease, had an ECOG performance status of 0–2, and had at least one measurable lesion according to RECIST 1.1. Patients were excluded if they had untreated symptomatic metastases in the central nervous system, a history of non-infectious pneumonitis that required corticotherapy, an active autoimmune disease, a positive HIV status, untreated active chronic hepatitis, a creatinine clearance less than 50 mL/min, or if they had used systemic corticosteroids (>10 mg prednisone or equivalent) or systemic immunosuppressive treatment within 14 days prior to the first dose of treatment. It is important to mention that patients who had previously received treatment for central nervous system (CNS) metastases and had a stable disease for a duration of more than 2 weeks were included.

This study was conducted in accordance with the Declaration of Helsinki and Good Clinical Practice guidelines and was approved by an independent ethics committee. All patients provided written informed consent, and all patient data were anonymized.

### 2.1. Procedures

Patients were chronologically included in three treatment cohorts (1A, 1B, and 2).

Cohort 1A: Patients were included from January 2016 until December 2017 and treated with a combination of nivolumab and ipilimumab following the protocol of the corresponding arm of the CheckMate-227 trial [3]. Patients first received nivolumab (360 mg intravenously every 3 weeks), followed by ipilimumab (1 mg/kg intravenously every 6 weeks). Treatment continued until disease progression (unless clinical benefit criteria were met for treatment beyond progression), unacceptable toxicity (grade 4 immune-related adverse events, or grade 3 immune-related adverse events that did not recover at grade 0–1 or where reintroducing immunotherapy could be medically dangerous for the patient in the opinion of the investigator), or completion per protocol (2 years).

Cohort 1B: Patients were included from January 2018 until December 2019 and treated with a combination of nivolumab and ipilimumab, along with a short-course chemotherapy regimen consisting of two cycles with a platinum doublet adapted to the histology, according to the CheckMate-9LA trial protocol [7]. Patients were first given nivolumab (360 mg intravenously every 3 weeks), followed by ipilimumab (1 mg/kg intravenously every 6 weeks). The chemotherapy treatment involved giving two cycles of carboplatin (AUC 6) and paclitaxel (200 mg/m^2^) to patients with squamous histology, whereas patients with non-squamous histology received carboplatin (AUC 5 or 6) and pemetrexed (500 mg/m^2^). Nivolumab, along with ipilimumab, was given until disease progression (except in cases where clinical benefit criteria were met for treatment beyond progression), until the treatment caused unacceptable toxicity (same definition as for cohort 1A), or until completion per protocol (2 years).

Cohort 2: Patients were included from January 2020 until June 2023 and treated with a combination of pembrolizumab and four cycles of platinum-based chemotherapy, according to the KeyNote-189 trial for non-squamous carcinomas [1] and KeyNote-407 trial for squamous carcinomas [8].

Patients were administered pembrolizumab at a dose of 200 mg every three weeks, for up to 35 cycles. For patients with squamous histology, the intravenous chemotherapy regimen consisted of four cycles of carboplatin (AUC 6) plus paclitaxel (200 mg/m^2^), while for patients with non-squamous histology, the regimen consisted of carboplatin (AUC 5 or 6) plus pemetrexed (500 mg/m^2^) for four cycles. Pembrolizumab was continued until disease progression or completion of 2 years or 35 cycles, whichever occurred first, unless the patient met the treatment beyond progression criteria or experienced unacceptable toxicity. Patients with non-squamous tumor histology received maintenance therapy with pemetrexed (500 mg/m^2^) until disease progression or unacceptable toxicity (same definition as for cohort 1A). In all cohorts, no dose reductions were allowed for pembrolizumab, nivolumab, or ipilimumab. Interruptions or discontinuations were conducted according to the data published for the CheckMate-227, CheckMate-9LA, KeyNote-189, and KeyNote-407 trials. In cohorts 1B and 2, chemotherapy dose reductions were allowed for toxicities, following local guidelines.

Tumors were evaluated initially and then every six weeks after the first dose for the first year, followed by an assessment every twelve weeks thereafter. The imaging methods did not change throughout the study period. The evaluation was carried out using CT with intravenous contrast of the thorax, abdomen, and pelvis. At least the thorax and upper abdomen were mandatory to be included in the evaluation. Patients underwent a baseline MRI of the brain, or CT of the brain if MRI was contraindicated. Patients with brain metastases were assessed according to standard care, usually every twelve weeks. A bone scan was performed at baseline and repeated every twelve weeks in patients with bone metastases. Adverse events were evaluated at the beginning of the treatment, during every scheduled visit, and whenever they happened during the treatment and the follow-up period, which lasted up to 100 days after the discontinuation of dosing. The severity of these events was graded using the National Cancer Institute Common Terminology criteria (version 4.0). Before starting the first cycle of treatment, patients underwent Hepatitis B and C testing within 28 days, HIV testing within 14 days, and thyroid tests within 14 days. Furthermore, hematology and chemistry tests were performed within 14 days before dosing, and on day 1 of every other cycle, including cycle one. Archival formalin-fixed, paraffin-embedded tissue samples were collected when available. The PD-L1 expression on viable tumor cells, represented as the Tumor Proportion Score (TPS), was evaluated using the approved immunohistochemical 28-8 PharmDx Assay (Dako) for nivolumab and the 22C3 PharmDx Assay (Dako) for pembrolizumab. Due to initial local difficulties in determining the PD-L1 status (lack of reimbursement), the determination of PD-L1 TPS score was recommended but not mandatory to include the patients in the treatment cohorts.

### 2.2. Outcomes

The main co-primary endpoints of this study were overall survival (OS), which is the time from the start of treatment until death from any cause, and progression-free survival (PFS), which is the time from the start of treatment until disease progression or death from any cause, whichever occurred first. The secondary objectives of the study were to determine the objective response rate (ORR) (confirmed complete response (CR) and partial response (PR) rates), as well as the clinical benefit rate (CBR) (CR, PR, and stable disease (SD) rates). Additionally, the study assessed the toxicity levels by recording any adverse events. A detailed analysis of the toxicity of different treatments was not completed in this report and is intended to be the subject of a future publication. Exploratory objectives included univariate and multivariate analysis of potential prognostic factors for objective response or clinical benefit, PFS, and OS, such as patient-related factors (age, gender, performance status), tumor-related factors (non-squamous vs. squamous histology, stage IVA vs. IVB, location of metastases), laboratory-related factors (hemoglobin, lymphocyte count, neutrophil count, neutrophil/lymphocyte ratio (NLR), platelets, LDH), treatment-related factors (best response obtained, duration of treatment, treatment after progression, rescue treatments—chemotherapy lines 2–4), and analysis of long-term survivors.

### 2.3. Statistical Analysis

Efficacy was determined in the intention-to-treat population, which included all patients who signed the informed consent. Toxicity was determined in patients who had at least one dose of the combination treatment. OS and PFS were computed from the first treatment day. The Kaplan–Meier method [13] was used to estimate OS and PFS. The significance level *p* was set to 0.05 for any statistical comparison and any interval confidence evaluation [14]. The squared chi test was used to compare the percentages, the Student t-test to compare averages, and the log-rank test to evaluate differences in OS and PFS. Multivariate analysis for the prognostic factors related to the clinical response/benefit was conducted using a logistic regression, and the multivariate analysis for OS and PFS was conducted using the Cox model [15]. A statistical comparison of OS and PFS between the treatments and the analysis of prognostic factors was limited to 2 years, due to the different follow-up in the three cohorts. Long-term survival data were available only for cohorts 1A and 1B.

## 3. Results

### 3.1. Patients and Treatment

Between January 2016 and June 2023, a total of 157 patients were enrolled during the screening period. Of these, 35 patients (21%) were excluded due to failure to meet the inclusion and exclusion criteria: untreated active hepatitis (10 of the 35 patients, 28.6%), untreated brain metastases (seven patients, 20%), creatinine clearance less than 50 mL/min (five patients, 14.3%), active autoimmune disease or corticotherapy >10 mg prednisone (or equivalent)/day within 14 days previous to the start of treatment (five patients, 14.3%), known EGFR/ALK actionable mutations or non-squamous histology with unknown EGFR/ALK status (four patients, 11.4%), or PS ECOG greater than 2 (four patients, 11.4%).

Out of the 122 included patients, 86 (70.5%) had non-squamous histology and 36 (29.5%) had squamous cell lung carcinoma. The patients were divided into three cohorts. Cohort 1A consisted of 18 patients who were enrolled in the study between 2016 and 2017. They were treated with a protocol used in the corresponding arm of the CheckMate-227 trial, which included nivolumab + ipilimumab. Cohort 1B had 33 patients who were enrolled between 2018 and 2019 and were treated like in the CheckMate-9LA trial, with nivolumab + ipilimumab plus two cycles of platinum-based chemotherapy. Of these, 25 patients had non-squamous tumors and 8 had squamous cell tumors. Cohort 2 included 71 patients who were enrolled between August 2019 and June 2023. They were treated with pembrolizumab plus four cycles of a platinum doublet. Of these, 52 patients had non-squamous tumors and were treated as in the KeyNote-189 trial, including maintenance pemetrexed. The remaining 19 patients had squamous tumors and were treated like in the KeyNote-407 trial.

In total, 41.8% of the patients were included in cohorts 1A and 1B and received the dual immunotherapy strategy, with or without short-course chemotherapy. The other 58.2% were included in cohort 2 and received mono-immunotherapy (pembrolizumab) combined with standard chemotherapy. Among all the treated patients, PD-L1 level was determined in only 55 patients (45.1%), due to local reimbursement constraints. Out of these, 26 (47.3% of the patients with known PD-L1 status) had negative PD-L1 levels (less than 1%) and 29 (52.7%) had positive PD-L1 levels (1% or more). As both PD-L1 positive and PD-L1 negative patients derived benefit in the combination immunotherapy trials, all patients were included in our cohorts, irrespective of the knowledge of the PD-L1 status. Baseline characteristics are presented in detail in Table 1. A separate column depicts the baseline characteristics for each of the three treatment cohorts. There were no significant differences in the baseline characteristics for the three treatment cohorts, with the exception of the number of metastases (*p* = 0.04, more patients with three or more metastatic sites in cohort 1B), the lymphocyte number (*p* = 0.03, with more patients with >1.5 × 10^3^/µL lymphocytes in cohorts 1A and 1B) and PD-L1 status (*p* < 0.01, with all patients evaluated for PD-L1 in cohort 1A, only 9.1% patients evaluated in cohort 1B, and 47.9% evaluated in cohort 2).

### 3.2. Overall Survival

As of 31 August 2023, database lock, the follow-up of patients was different, depending on the period of time in which the patients were successively enrolled in the three cohorts, specifically when the different immunotherapy drugs became available for research and clinical use in our Institute (Table 2).

**Figure 1:**
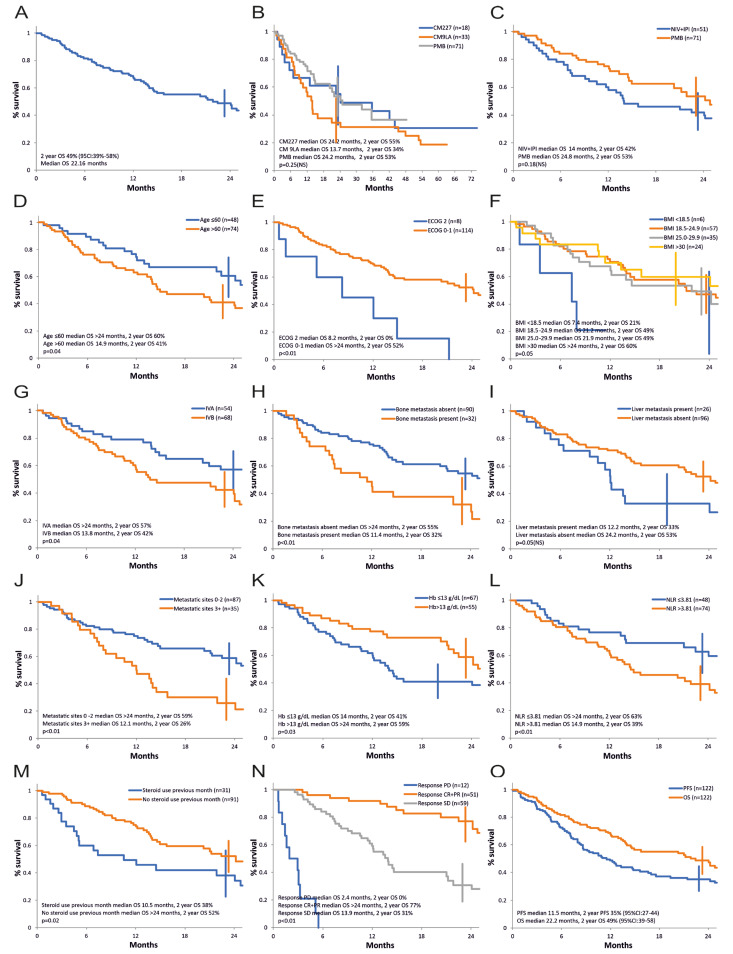
Overall survival, univariate analysis of prognostic factors. (**A**) All patients. (**B**) According to treatment protocol (CM227—CheckMate-227 protocol, cohort 1A; CM9LA—CheckMate-9LA protocol, cohort 1B; PMB—pembrolizumab, KeyNote-189 and KeyNote-407 protocol, cohort 2). (**C**) Immunotherapy strategy (NIV + IPI—nivolumab and ipilimumab, CheckMate-227 and CheckMate-9LA protocol, cohort 1A and cohort 1B; PMB—pembrolizumab, KeyNote-189 and KeyNote-407 protocol, cohort 2). (**D**) Age. (**E**) ECOG PS—Eastern Cooperative Oncology Group Performance Status. (**F**) BMI—Body Mass Index. (**G**) AJCC stage. (**H**) Bone metastasis. (**I**) Liver metastasis. (**J**) Number of metastatic sites. (**K**) Hemoglobin level. (**L**) Neutrophil-to-lymphocyte ratio. (**M**) Steroid use in the first month. (**N**) Response to treatment. (**O**) OS and PFS, all patients.

The overall survival curve of patients in our series is shown in Figure 1. The median survival with the different immunotherapy combinations for advanced NSCLC was 22.2 months, and the survival rate at 24 months was 49% (95%CI: 39–58%), Figure 1A.

The survival curve according to the therapy protocol is presented in Figure 1B. Median survival in cohorts 1A vs. 1B vs. 2 was 24.2 vs. 13.7 vs. 24.2 months and actuarial 2-year survival was 55% vs. 34% vs. 53%. The differences were not statistically significant (*p* = 0.25). The analysis considered the different follow-up times of each cohort and only data from cohorts 1A and 1B are available at this moment for long-term survival.

Figure 1C compares the strategy of nivolumab + ipilimumab (cohort 1A and 1B) versus pembrolizumab (cohort 2) at a 2-year follow-up. Numerically, there is an advantage for pembrolizumab therapy. Median survival is 24.8 months for pembrolizumab versus 14 months for nivolumab + ipilimumab and the survival rate at 2 years is 53% and 42%, respectively. However, this difference does not reach statistical significance (*p* = 0.18, HR 0.71, 95% CI 0.62–0.8).

Summarizing the results of the univariate analysis in terms of prognostic factors for overall survival, a demonstrated negative statistical significance was found for age over 60 years, PS ECOG 2, BMI < 18.5 (but the number of patients in this category was small), stage IVB, the presence of bone metastases, the presence of three or more metastatic sites, Hb ≤ 13 g/dL, neutrophil/lymphocyte ratio (NLR) over 3.81, treatment with high doses of corticotherapy in the first month of therapy, as well as progressive or stable disease as best response compared to obtaining an objective response (Figure 1D–H,J–N, Table 3). The presence of liver metastases had a marginal statistical influence (*p* = 0.05, Figure 1I, Table 3).

Factors such as gender, smoking status, histology, presence of pulmonary, pleural, CNS, adrenal metastases or metastases in other sites, PD-L1 expression level, presence of actionable mutations (KRAS G12C, cMET, RET, and, discovered post-immunotherapy progression EGFR, ALK), level of neutrophils, lymphocytes, platelets, LDH, previous recent palliative radiotherapy, as well as treatment strategy or individual treatment protocol did not significantly influence survival in the univariate analysis in our series (Table 3). It is worthy of note that the analysis of the prognostic value of PD-L1 and LDH could not be optimally performed, as 67 patients (54.9%) had an unknown PD-L1 expression and 34 patients (27.9%) had an unknown initial LDH value.

A multivariate analysis using the Cox model for overall survival was carried out and included the baseline factors identified in the univariate analysis, ungrouped (age at study entry, ECOG status, BMI, AJCC stage, presence of bone metastases, liver metastases, number of metastases, hemoglobin value, neutrophil to leukocyte ratio, steroid use in the first month). Two independent variables were retained: age at study entry (*p* = 0.03, HR 1.03) and ECOG PS status (*p* = 0.04, HR 2.08).If in the multivariate analysis, we excluded the number of metastases as being possibly not independent of the site of metastases, four independent unfavorable prognostic factors were retained: older age at study entry (*p* = 0.02, HR 1.03, 95% CI 1.004 to 1.07), ECOG PS 2 vs. 0–1 (*p* = 0.02, HR 2.17 95% CI 1.08 to 4.36), neutrophil/leukocyte ratio (>3.81 vs. ≤3.81) (*p* = 0.03, HR 1.81, 95% CI 1.04 to 3.15), and steroid use in the first month (*p* = 0.04, HR 1.79, 95% CI 1.01 to 3.16).

### 3.3. Progression-Free Survival

**Figure 2:**
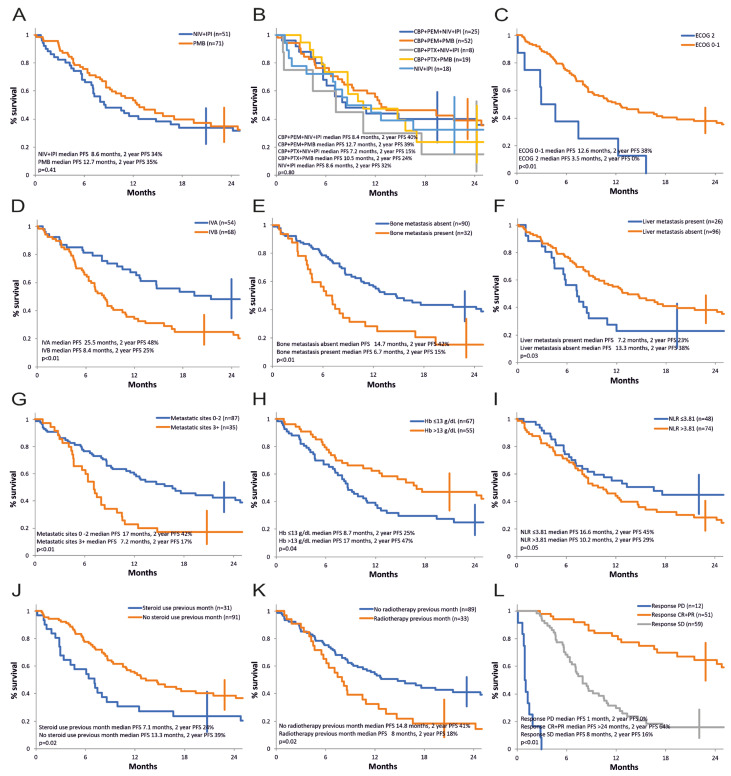
Progression-free survival, univariate analysis of the prognostic factors. (**A**) Immunotherapy strategy (NIV + IPI—nivolumab and ipilimumab, CheckMate-227 and CheckMate-9LA protocol, cohort 1A and cohort 1B; PMB—pembrolizumab, Keynote-189 and Keynote-407 protocol, cohort 2. (**B**) Treatment protocol (CBP—carboplatin, PEM—pemetrexed, PTX—paclitaxel, NIV—nivolumab, IPI—ipilimumab; CBP + PEM + NIV + IPI—Cohort 1B non-squamous; CBP + PEM + PMB—Cohort 2 non-squamous; CBP + PTX + NIV + IPI—Cohort 1B squamous; CBP + PTX + PMB—Cohort 2 squamous; NIV + IPI—Cohort 1A). (**C**) ECOG PS. (**D**) AJCC stage. (**E**) Bone metastasis. (**F**) Liver metastasis. (**G**) Number of metastatic sites. (**H**) Hemoglobin level. (**I**) Neutrophil-to-lymphocyte ratio. (**J**) Steroid use in the first month. (**K**) Radiotherapy use in previous month. (**L**) Response to treatment.

The progression-free survival curves are shown in Figure 1O and Figure 2. Median PFS for advanced NSCLC was 11.5 months, and 24-months progression-free survival rate was 35% (CI: 27–44%) (Figure 1O). Figure 2A compares the strategy with nivolumab + ipilimumab versus pembrolizumab at 2-year follow-up. Numerically, there is an advantage for pembrolizumab therapy. Median PFS is 12.7 months for pembrolizumab versus 8.6 months for nivolumab + ipilimumab, but progression-free survival rate at 2 years is quasi-identical (35% vs. 34%). The difference between the PFS curves does not reach statistical significance (*p* = 0.41, HR = 0.83 limits 0.75–0.92). We observe a numerically longer PFS for pembrolizumab protocols (median PFS 12.7 months and 2-year actuarial PFS 39% for KN-189-like; 10.5 months and 24% for KN-407; 8.6 months and 32% for CM-227; 8.4 months and 40% for CM-9LA non-squamous; and 7.2 months and 15% for CM-9LA squamous) with no statistical significance between them in our series (see Figure 2B).

Summarizing the results of the univariate analysis, a negative statistical significance for PFS was demonstrated for PS ECOG 2, stage IVB, presence of bone metastases, liver metastases, presence of three or more metastatic sites, Hb ≤ 13 g/dL, neutrophil/lymphocyte ratio (NLR) > 3.81, high-dose corticosteroid treatment in the first month of therapy, recent prior palliative radiotherapy, and progressive and stable disease as best response versus achieving an objective response (Table 3, Figure 2C–L.)

There was no prognostic significance in our series for age, gender, BMI, smoking status, histology, lung, pleural, CNS, adrenal, other site metastases, PD-L1 expression level, presence of actionable mutations (KRAS G12C, cMET, RET, EGFR, ALK—the latter two found in post-immunotherapy analysis), neutrophil, lymphocyte, platelet levels, LDH value, and treatment strategy (nivolumab + ipilimumab vs. pembrolizumab combinations) or individual treatment protocol (data at 2 years), as detailed in Table 3. As for the OS analysis, the prognostic value of PD-L1 and LDH for PFS could not be optimally performed, as 67 patients (54.9%) had an unknown PD-L1 status and 34 patients (27.9%) had an unknown initial LDH value.

There are some differences between the results of the univariate analysis of prognostic factors for overall survival and progression-free survival. Age and BMI (below 18.5) were significant only for OS, and previous recent palliative radiotherapy was significant only for PFS.

A multivariate analysis using the Cox model for progression-free survival was carried out and included the eight baseline factors identified in the univariate analysis (ECOG status, AJCC stage, bone metastases (Yes/No), liver metastases (Yes/No), number of metastases (<3 vs. ≥3), hemoglobin value (≤13 g/dL vs. >13 g/dL), neutrophil/leukocyte ratio (≤3.81 vs. >3.81), corticotherapy in the first month of treatment (Yes/No). Only one independent variable was retained: ECOG status (*p* = 0.02, HR 2.03, 95% CI 1.08 to 3.79). If in the multivariate analysis, we excluded the number of metastases as being possibly correlated with the site of metastases, performance status ECOG was again the only independent factor retained (*p* = 0.02, HR 2.04, 95% CI 1.09 to 3.81).

### 3.4. Tumor Response

Objective responses (CR + PR) were obtained in 51 of the 122 patients (41.8%) including one complete response (0.8%) and 50 partial responses (41%). Stable disease (SD) was noted in 59 patients (48.4%). Clinical benefit (CR + PR + SD) was achieved in 110 patients (90.2%). The remaining 12 patients (9.8%) had disease progression (PD), as assessed by the radiological team from our institution.

Univariate analysis of prognostic factors for obtaining an objective response (CR + PR) is presented in Table 4.

Following univariate analysis, performance status 2 and high-dose corticosteroid therapy (>10 mg/prednisone/day) in the first month of treatment were identified as unfavorable prognostic factors for obtaining an objective response (for both, *p* = 0.03).

Univariate analysis of prognostic factors for achieving clinical benefit (CR + PR + SD) is also detailed in Table 4. The following prognostic factors were identified as unfavorable at baseline for obtaining a clinical benefit (CR + PR + SD): age ≥ 61 years (*p* = 0.04), ECOG PS 2 (*p* < 0.01), absence of lung metastases (*p* < 0.05), hemoglobin ≤ 13 g/dL (*p* = 0.04), neutrophils to lymphocytes ratio > 3.81 (*p* = 0.04), and steroid use (>10 mg/prednisone/day or equivalent) in the first month of treatment (*p* = 0.02).

**Figure 3:**
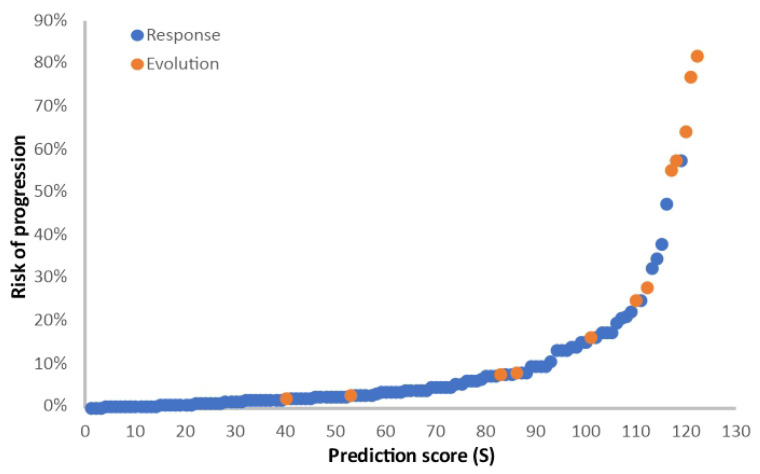
Logistic model for the analysis of clinical benefit. Prediction score (S) for the risk of progressive disease, calculated as S = 0.152 × age + 2.6055 × ECOG PS + 2.2167 × corticotherapy (1 for yes and 0 for no). Blue dots, patients with clinical benefit (CR + PR + SD); orange dots, patients with progressive disease (PD).

A multivariate analysis related to clinical benefit was carried out and included the six factors identified in the univariate analysis (age at study entry, ECOG status, pulmonary metastases present, hemoglobin value, neutrophil/leukocyte ratio, corticotherapy in the first month). The multivariate analysis using the logistic model retained three independent unfavorable prognostic factors: ECOG PS (2 vs. 0–1) (OR 12, 95% CI 1.59–90.35, *p* = 0.01), use of corticotherapy in the first month of the treatment (OR 9.56, 95% CI 1.75–52.13, *p* < 0.01), and age ≥ 61 (OR 1.17, 95% CI 1.03–1.34, *p* = 0.012). The prognostic score is S = 0.152 × age + 2.6055 × ECOG PS + 2.2167 × corticotherapy (1 for yes and 0 for no). The risk of progressive disease with immunotherapy is Exp(S)/(1 + Exp(S)). The higher the score, the higher the risk of progressive disease, as presented in Figure 3.

### 3.5. Treatment beyond Progression

In 21 patients (representing 17.2% of the total 122 patients enrolled in the study), first-line therapy was continued beyond progression according to RECIST criteria in patients with clinical benefit who so desired. In 6 of the 21 (28.6%), a temporary stabilization was achieved, and in 15 (71.4%) the disease continued to progress.

### 3.6. Subsequent Treatments

Subsequent second line systemic therapy after progression on first-line immunotherapy combinations was received by 48 (39.3%) of the 122 patients. Second line treatments consisted of chemotherapy with a platinum doublet (44 patients), or targeted molecular therapy (4 patients). Third line systemic treatment was received by 21 patients (17.2%) and consisted of chemotherapy (docetaxel, 18 patients) and targeted therapy (3 patients). Fourth line systemic treatment was received by seven patients (5.7%) and consisted of chemotherapy (gemcitabine or vinorelbine, six patients) or immunotherapy (one patient).

### 3.7. Long-Term Survivors

At the time of the present analysis (1.SEP.2023), 80 of the 122 patients had a follow-up of at least 48 months. Among the 80 patients, 14 (17.5%) were alive at ≥48 months. There were no long-term survivors in patients with baseline PS = 2, body mass index < 18.5, and patients with bone metastases. There were significantly more 4-year survivors in patients with stage IVA vs. IVB (28.6% vs. 8.9%, *p* = 0.02), NLR ≤ 3.81 vs. > 3.81 (32.3% vs. 8.2%, *p* < 0.01). There was no correlation between long-term survival and gender, age (≤60 vs. >60), presence of liver metastases (yes vs. no), number of metastases (<3 vs. ≥3), hemoglobin value (≤13 g/dL vs. >13 g/dL), or corticotherapy in the first month of treatment (yes vs. no).

Our long-term data come mainly from cohorts 1A (18 patients, median follow-up 83 months, range 77.8–84.4 months) and 1B (33 patients, median follow-up 59 months, range 49.1–63 months).

In cohort 1A, we noticed a 30.5% actuarial 4-year and 5-year survival (95% CI 14–54%), initiating a plateau in survival. In the CheckMate-227 study, with a minimal follow-up of 49.4 months and a median follow-up of 54.8 months (range 49.4–65.8 months) in the corresponding nivolumab/ipilimumab arm, the actuarial 4-year survival was 29% in the PD-L1-expressing patients and 24% in the PD-L1 < 1% subset of patients.

In cohort 1B we noticed a 25% (95% CI 13–43%) actuarial 4-year survival and a 18.8% (95% CI 9–35%) 5-year survival. In the corresponding nivolumab/ipilimumab + short course chemotherapy arm from the CheckMate-9LA study, with a median follow-up of 47.9 months, 4-year OS was 21%.

For the dual immunotherapy cohorts, long-term survival in our series was in the same range as the one obtained in the registration trials using similar treatment regimens.

Cohort 2 (71 patients) had a shorter median follow-up of 14.2 months (range 2.9–48.3 months), with a promising 53% 2-year actuarial survival. Longer follow-up is needed to report the long-term survivors’ data. In the updated analysis of the phase 3 KeyNote-189 study, for the corresponding chemotherapy + pembrolizumab arm, 4-year OS was 28.3% and 5-year OS was 24.7% (95% CI 19.6–30.2%).

### 3.8. Toxicity

A more detailed description of the toxicity, corresponding to the different treatment protocols, will be the subject of another publication. In brief, the toxicity of treatments, including immunotherapy, was moderate, with hematological toxicity occurring mainly during the combination with the chemotherapy sequence. Toxicity may be underestimated under real-world conditions, which are less stringent in terms of toxicity data collection. Among the hematological toxicities, the most common was anemia (20.5%), followed by thrombocytopenia (6.4%) and neutropenia (3%). One case of thrombocytopenia had a fatal outcome. Among the toxicities in which the autoimmune mechanism can be blamed, liver toxicity was the most common (11.5%), followed by pneumonitis (8.2%). Of the 10 cases of pneumonitis, one had a fatal outcome. The other autoimmune toxicities were endocrine, cutaneous, renal, pancreatic enzymes elevation (6.6% each), diarrhea (5.7%), ocular toxicity (4%), colitis (1.6%), and cardiac toxicity (1.6%).

The occurrence of toxicity led to temporary interruption of therapy until resolution of grade 1 or 0 toxicity in 11 patients (9% of cases) and led to permanent discontinuation of immunotherapy in 19 patients (15.6% of cases).

## 4. Discussion

### 4.1. Landscape of the Immunotherapy Combinations

In the last five to ten years, the standard of care for first-line treatment of advanced NSCLC without actionable driver mutations has shifted from chemotherapy alone to immunotherapy-based regimens. Patients with high PD-L1 expression may benefit from immunotherapy alone and OS results are similar for mono-immunotherapy and immunotherapy–chemotherapy combinations. However, PFS and ORR results favor the combinations, as shown in a meta-analysis by Wang et al. [16].

Patients may benefit from immunotherapy combinations irrespective of their PD-L1 expression. To our best knowledge, a direct comparison between these strategies was not performed. We looked at three different combination modalities (cohort 1A: nivolumab + ipilimumab, cohort 1B: nivolumab + ipilimumab + short course of a platinum doublet, and cohort 2: pembrolizumab + full course of a platinum doublet chemotherapy) that were used in our institution in consecutive time periods. Due to the study design, a head-to-head comparison was not possible; however, a non-randomized comparison was conducted at a 2-year landmark for the three cohorts. Long-term data were available only for cohorts 1A (median follow-up 83 months) and 1B (median follow-up 59 months), due to the shorter follow-up for cohort 2 (14.2 months).

### 4.2. Rationale for the Immunotherapy Combinations—The Complementary Mechanisms of Action

Anti-PD-1 antibodies and anti-CTLA-4 antibodies are immune checkpoint inhibitors with distinct but complementary mechanisms of action. Anti-PD-1 agents act in the effector phase of the immunity, in the tumor and microenvironment battlefield, when they re-activate the exhausted effector T-cells and enhance the preexisting T-cell response. Anti-CTLA-4 agents act in the priming phase of the immunity in the lymph nodes, induce T-cell proliferation, drive de novo antitumor T-cell responses, including an increase in memory T-cells, and overcome the immune suppression of T-regs. Both anti-PD-1 and anti-CTLA4 agents increase cytokine production and alter the microenvironment towards a pro-inflammatory state [7,17,18,19]. Nivolumab plus ipilimumab is indicated in the USA and other countries for the first-line treatment of adults with metastatic NSCLC with PD-L1 ≥ 1% and no EGFR or ALK genomic tumor aberrations. However, a similar benefit was observed in the CheckMate-227 study for patients with PD-L1 < 1%, based on a descriptive analysis. In our study, inclusion of patients in cohort 1A was permitted irrespective of the PD-L1 status.

The addition of chemotherapy in the CheckMate-9LA study (corresponding to cohort 1B in our series) was conducted with the aim to provide early disease control in the first few weeks of therapy and the short course (two cycles) was intended to minimize the side-effects that are associated with a full course of chemotherapy [7]. 

The combination of an anti-PD-1 agent and a full course of chemotherapy (as used in the KeyNote-189 and KeyNote-407 trials and in cohort 2 in our series) is probably the most used modality of immunotherapy combination today. Chemotherapy can lead to immunogenic cell death, and thus promote the activation of antigen-presenting cells (APCs) by releasing tumor cell fragments. As a result, more activated APCs migrate in the priming phase to the lymph nodes via blood and lymph vessels, leading to the activation of more tumor-specific T-cells and enhancing the immune response. Negative immunoregulatory cells (such as T_reg_ cells) can also be eliminated by chemotherapy [20,21,22,23,24].

### 4.3. Purpose and Limitations of the Study

We intended to build our own experience on different protocols, as they were chronologically available in our institution, keeping the inclusion/exclusion and diagnostic procedures for the successive treatments as close as possible. An analysis of the efficacy of the different combinations was conducted in the different cohorts.

The limitations of this study are related especially to the relatively small number of patients included (n = 122), as the study reflects the experience of a single institution. The comparison between the protocols is also limited by the fact that the study was not randomized; the patients were included in the different cohorts successively, as the different immunotherapy agents became available. Due to the local non-availability of the testing at the beginning of the study, PD-L1 status was performed only in 55 of the total 122 patients (45.1%). LDH baseline value was not available in 34 patients (27.9%). Therefore, the analysis of the prognostic value of both PD-L1 and LDH is not optimal in our series.

### 4.4. Overall Survival, Progression-Free Survival, and Long-Term Survivors

In our series of 122 patients, median survival was 22.2 months and actuarial survival at 24 months was 49% (CI: 39–58%). These data are comparable to the best published results in the literature. The KeyNote-189 study [1] reports a median survival of 22 months and an actuarial survival of 45.7% at 2 years for non-squamous carcinomas; the KeyNote-407 study [8] finds a median survival of 17.2 months and actuarial survival of 36% at 2 years for squamous cell carcinomas; the CheckMate-227 study [3] reports a median survival of 17.1 months and actuarial survival of 40% at 2 years, and the CheckMate-9LA study [7] finds a median survival of 15.6 months and actuarial 45% at 2 years, all histologies together.

The progression-free survival analysis found a median PFS of 11.5 months and actuarial progression-free survival at 24 months of 35% (CI: 27–44%). These data are also comparable to the best published results in the literature. The KeyNote-189 study [1] reports median PFS of 9 months and actuarial survival of 23.1% at 2 years for non-squamous carcinomas; the KeyNote-407 study [8] reports a median PFS of 8 months and actuarial survival of 20.7% at 2 years for squamous carcinomas; and the CheckMate-9LA study [7] reports a median PFS of 6.7 months and actuarial survival of 20% at 2 years.

The comparison of the overall survival for the two combining strategies was conducted at a 2-year follow-up. Median survival was 24.8 months for cohort 2 versus 14 months for cohorts 1A and 1B, and actuarial survival at 2 years is 53% and 42%, respectively. However, this difference does not reach statistical significance (*p* = 0.18, HR = 0.71, limits 0.62–0.8).

The comparison of the two strategies (cohort 2 vs. cohorts 1A and 1B) in terms of PFS found a median of 12.7 months for pembrolizumab versus 8.6 months for nivolumab + ipilimumab, and actuarial progression-free survival at 2 years was quasi-identical (35% vs. 34%). The difference between the PFS curves did not reach statistical significance (*p* = 0.41, HR = 0.83 limits 0.75–0.92).

A conclusion in terms of choosing the best combination strategy cannot be formulated from our series, taking into account the statistical limitations previously described. Cohort 2 had numerically better results for OS and PFS at 2 years, not reaching statistical significance.

However, with an 83- and 59-month median follow-up for cohorts 1A and 1B, respectively, we could confirm the existence of long-term survivors, many of them without treatment and without relapse for 2 to 5 years, after having finished the first-line dual immunotherapy treatment.

Cohort 1A (18 patients) has a median follow-up of almost 7 years, permitting us to observe 30.5% long-term survivors, with a plateau of survival starting at 4 years of follow-up.

Cohort 1B (33 patients) has a median follow-up of almost 5 years (59 months), with 18.8% alive at 5 years.

Cohort 2 (71 patients) has the shortest follow-up (median 14.2 months). The results at 2 years are at least as good as for the cohort 1 patients. No long-term results are yet available.

### 4.5. Univariate and Multivariate Analysis of the Prognostic Factors

This study allowed for extensive univariate analysis for OS, PFS, and clinical benefit, with applications to the use of immunotherapy combinations in clinical practice (e.g., negative impact on prognosis for older age, PS = 2, stage IVB, anemia, NLR > 3.81, use of corticoids early in the treatment; blunting of prognostic differences related to histology and PD-L1 expression level; usefulness of immunotherapy even in patients with a reserved prognosis with brain or liver metastases).

The three separate multivariate analysis performed with the Cox model for OS and PFS and with the logistic model for obtaining a clinical benefit found:

- Four independent prognostic factors for OS (unfavorable being deteriorated ECOG performance status (2 vs. 0–1) (*p* = 0.02, OR 2.17, 95% CI 1.08 to 4.36), older age at study entry (*p* = 0.02, OR 1.03, 95% CI 1.004 to 1.07), use of corticotherapy in the first month of the treatment (yes vs. no) (*p* = 0.04, OR 1.79, 95% CI 1.01 to 3.16), neutrophil/leukocyte ratio (>3.81 vs. ≤3.81) (*p* = 0.03, OR 1.81, 95% CI 1.04 to 3.15).
- One independent prognostic factor for PFS: ECOG status (2 vs. 0–1) (*p* = 0.02, OR 2.03, 95% CI 1.08 to 3.79).
- Three independent prognostic factors for obtaining a clinical benefit: ECOG PS (2 vs. 0–1) (OR 12, 95% CI 1.59 to 90.35, *p* = 0.015), age (≥61 vs. <61) (OR 1.17, 95% CI 1.03 to 1.34, p = 0.01), and use of corticotherapy in the first month of the treatment (yes vs. no) (OR 9.56, 95% CI 1.75 to 52.13, *p* = 0.009).

### 4.6. Performance Status

Performance status is generally considered as an important prognostic factor for the success of cancer treatments in general. Although the registration first-line trials for checkpoint inhibitors (pembrolizumab, atezolizumab, nivolumab) included only patients with PS = 0–1, these drugs are approved and used in real-world practice regardless of the performance status of the patients [25,26,27]. Due to their lower toxicity profile in comparison with chemotherapy, the administration of immunotherapy (alone or in combination) is attractive in real-world settings both for patients with worse performance status or who are older.

A few immunotherapy studies included patients with PS = 2: 5 patients treated with pembrolizumab (<1% of the study population) in KeyNote-10 [28], 128 patients treated with nivolumab (=9% of the study population) in CheckMate-153 [29], 103 patients treated with nivolumab (=12.7%) in CheckMate-171 [30], and 139 patients (=23.6%) treated with nivolumab + ipilimumab in CheckMate-817 [31]. Median overall survival was shorter in patients with an ECOG PS = 2 compared with the overall study population: 4 vs. 9.1 months in CheckMate-153, 5.2 vs. 10 months in CheckMate-171. Similarly, median PFS was shorter in PS = 2 patients, 3.6 vs. 6 months in CheckMate-817.

In our study, PS = 2 patients represented 6.6% of the 122 patients. With the limitation represented by this small number, they had a worse outcome for all the three efficacy measures (OS, PFS, and clinical benefit).

### 4.7. Age

Data related to the negative prognostic significance of older age [25] seem controversial in studies related to checkpoint inhibitors.

Advanced age alone does not predict for a lack of clinical benefit in most previous studies with cytotoxic chemotherapy, but a higher risk of chemotherapy toxicities was found [32,33]. Given a more favorable toxicity profile, it is reasonable to consider that immunotherapy with PD-1/PD-L1 inhibitors would be an acceptable treatment regimen for the elderly. There are a limited number of immunotherapy clinical trials dedicated to the elderly, potentially putting such patients at risk for undertreatment and overtreatment biases [34]. For immunotherapy, subgroup analyses (≥65 vs. <65 years old) from three registration trials have shown no difference [1,8] or a decreased survival benefit for elderly patients (≥65 years old) [35].

In the single-agent CheckMate-153 and CheckMate-171 trials [29,30], where nivolumab alone was given as rescue treatment and which included 556 patients (representing 39% of the total number) and 278 patients (34%), respectively, aged ≥70 years, median overall survival time was comparable in the overall population and the older patients. Similar results come from a retrospective study from Wake Forrest University in Winston-Salem [25], where patients with advanced NSCLC treated with immunotherapy had an OS, PFS, and overall response that did not differ significantly by age group.

We also observe that in CheckMate-153, CheckMate-171, and the Wake Forrest study, safety was similar across age subgroups.

On the other hand, the registration trials for different immunotherapy combinations versus standard chemotherapy have all found less benefit for the experimental arm in terms of OS for older populations [1,3,7,8]. The hazard ratios for the dual nivolumab + ipilimumab immunotherapy versus chemotherapy for the subsets of patients <65 years old vs. ≥65 to <75 years old vs. ≥75 years old were the following: CheckMate-227, 0.7 vs. 0.91 vs. 0.92 [3]; CheckMate-9LA, 0.61 vs. 0.62 vs. 1.21 [7]. The hazard ratios for pembrolizumab + chemotherapy versus chemotherapy for the subsets of patients <65 years old vs. ≥65 years old were the following: KeyNote-189, 0.43 vs. 0.64 [1]; KeyNote-407, 0.52 vs. 0.74 [8].

In our study, 39 of the 122 patients (32%) were >65 years old and in the multivariate analysis, older age had a negative impact on OS and clinical benefit. These results are in line with those previously reported with the combination immunotherapy studies. In summary, the data in the literature suggest that immunotherapy combinations improve prognosis over chemotherapy at all ages, but the impact is less with older age. In the case of mono-immunotherapy, older age does not seem to impact efficacy results. The discrepancy is possibly due to the more important toxicity of the immunotherapy combinations in elderly patients, compared with the mono-immunotherapy studies.

### 4.8. Corticosteroids

Corticosteroids are mostly used in oncology for their anti-inflammatory effects to help with pain and other symptoms, their anti-cancer effects in some types of blood cancer, their ability to lower the immune system to deal with immune-related adverse events (irAEs) caused by immunotherapy, and their role for allergy or emesis prophylaxis for different cancer treatments [36]. Some of the effects of corticotherapy are immunosuppressive [37], and therefore they are the mainstay of the treatment of the autoimmune effects of the checkpoint inhibitors. The administration of corticosteroids at the initiation of checkpoint inhibitors or at a later stage of the therapy could compromise the development of an immune response.

It was reported that 14% to 33% of individuals diagnosed with non-small cell lung cancer who begin treatment with immune checkpoint inhibitors are prescribed doses of prednisone or equivalent above 10 mg/day [38,39]. Two separate studies conducted at Institut Gustave Roussy, Villejuif (with a sample size of 185) and Memorial Sloan Kettering Cancer Center (with a sample size of 455) revealed that the use of corticoids with checkpoint inhibitors resulted in worse overall response rate (ORR), progression-free survival (PFS), and overall survival (OS). The use of corticosteroids at the beginning of treatment resulted in a deterioration of progression-free survival (HR 1.3, *p* = 0.03) and overall survival (HR 1.7, *p* < 0.001) according to the multivariate analysis [38,39]. A study including 210 patients found that those who were administered corticosteroids during the first month of therapy with nivolumab (n = 25) had a significantly lower median overall survival (OS) compared to those who did not receive corticosteroids (4.3 months vs. 11 months; *p* = 0.006) [38]. In a study by Arbour et al., the use of corticosteroids at baseline resulted in decreased OS (HR, 1.7; *p* < 0.001) and PFS (HR, 1.3; *p* = 0.03) [39]. A similar outcome was found by Fuca et al. in metastatic NSCLC, where the early use of steroids impaired OS (HR, 2.60; *p* = 0.001), PFS (HR, 1.80; *p* = 0.003) and disease control (odds ratio, 0.32; *p* = 0.006) [40].

According to a systematic review and meta-analysis by Petrelli et al. [41] that covered 16 studies including 4045 patients, those having steroids were at increased risk of death (HR 1.54, 95% CI 1.24–1.91; *p* = 0.01) and progression (HR 1.34, 95% CI 1.02–1.76; *p* = 0.03), compared with the patients not treated with corticosteroids. Using corticotherapy to treat the adverse events of immunotherapy did not impair OS, but survival was worse if corticoids were used for symptom control.

A study conducted by Ricciuti et al. [42] in 650 patients with NSCLC who were undergoing immunotherapy found that patients who were treated with a dose of prednisone >10 mg/day at the start of immunotherapy had worse outcomes compared to patients who received a dose between 0 and 10 mg. The median overall survival time was 4.9 months for the high-dose group and 11.2 months for the low-dose group (*p* < 0.001).

A retrospective analysis was conducted on 424 patients with advanced NSCLC who were treated with a single checkpoint inhibitor [42]. Among these patients, 49 individuals were administered steroids during the first 8 weeks of treatment. Patients undergoing steroid treatment for cancer-related symptoms had a significantly shorter median overall survival time of 1.9 months compared to those who received corticosteroids for non-palliative reasons, such as immune-related adverse events and chronic obstructive pulmonary disease, who had a median overall survival time of 13.4 months. 

In line with the previous observations, the use of corticotherapy equivalent to >10 mg/day of prednisone in the first month of treatment had an independent negative impact for overall survival (HR 1.79, 95% CI 1.01–3.16, *p* = 0.04), and for obtaining a clinical benefit (HR 9.56, 95% CI 1.75–52.13, *p* = 0.009), in our series. The short use of corticotherapy recommended for the administration of certain drugs (taxanes or pemetrexed) or as part of the antiemetic prophylaxis was not taken into account.

On the other hand, a systematic review by Garant et al. [43] and a study from Tata Memorial Hospital [44] concluded that the concomitant administration of corticosteroids and checkpoint inhibitors may not necessarily lead to poorer clinical outcomes.

Putting all these somewhat controversial results together, we think it is reasonable to consider that early steroid use interacts with the building of an immune response, and therefore may reduce the activity of the checkpoint inhibitors, which may need an amount of time to act. Some studies, including our series, found a significant correlation between early use of corticotherapy and a worse outcome. This probably reflects the worse prognosis of those patients who needed corticoids to palliate their cancer symptoms. On the other hand, later use of corticosteroids in a non-palliative setting, to treat the immune-related adverse events, did not seem to modify the response to checkpoint inhibitors [36].

### 4.9. Neutrophil-to-Lymphocyte Ratio

Neutrophil-to-lymphocyte ratio (NLR), a factor that proved prognostic for OS in our study, was evaluated as an emerging prognostic marker in different disease fields, such as sepsis, COVID-19, cardiovascular diseases, and cancer, and generally proved to be a cheap and useful prognostic clinical marker; however, there has been no clear threshold established in the different settings [45].

A rise in the NLR may be the result of an isolated rise in the neutrophil count and may occur in situations related to tissue damage that activate a systemic inflammatory response. It can be observed in cancer but also infections (bacterial or fungal), myocardial infarction, stroke, atherosclerosis, trauma, and surgical complications. In the general population, an elevated NLR could predict mortality [45,46,47]. Tumor initiation switches on the ”cancer elicited inflammation” [48]. The resulting tumor-associated neutrophils (TANs) and tumor-associated macrophages (TAMs) may increase the systemic neutrophilic inflammation [48,49] and produce metastatic progression.

Lymphocytes, including B-cells, T-cells (T4 positive, CD4/CD8 negative, or CD8 positive), and natural killer T-cells, play a crucial role in adaptive immunity by generating a targeted response to particular antigens via the involvement of MHC class I and II molecules. A decreased neutrophil-to-lymphocyte ratio (NLR) indicates a maintained immunological equilibrium and is often linked to a positive prognosis in all areas of use [48,49].

Different studies in oncology generally agreed on the prognostic value of NLR in cancer, but a clear threshold is controversial. Our study confirmed the prognostic role of baseline NLR for OS and found a threshold of 3.81 for patients with advanced NSCLC treated with immunotherapy combinations. In urothelial cancer treated with immunotherapy, cut-offs ≥3 and ≥5 predicted progressive disease and poorer PFS and OS [50]. In hepatocarcinoma, a rising NLR was also found as prognostic. In glioblastoma, a cut-off NLR < 4.7 predicted a longer PFS. A NLR higher than 4.95 was correlated with the presence of brain metastases in NSCLC, at baseline or during follow-up [51]. A meta-analysis by Templeton et al. [49] showed that a NLR > 4 predicts a shorter overall survival with different tumors, and also a shorter cancer-specific survival, PFS, and DFS.

## 5. Conclusions

In our series, in the first-line systemic treatment of advanced NSCLC, combining an anti-PD-1 checkpoint inhibitor with (a) another immunotherapy (anti-CTLA4) with or without a short course chemotherapy with a platinum doublet or with (b) a full course platinum doublet chemotherapy (plus pemetrexed maintenance for non-squamous NSCLC) lead to an overall survival and progression-free survival similar to those observed in the registration studies and represented a clear historical advance over chemotherapy alone. Median OS and PFS for all patients were 22.2 and 11.5 months and 2-year actuarial OS and PFS were 49% and 35% in the intent-to-treat population.

The two immunotherapy combination strategies have achieved similar results at two years in our series, in terms of OS and PFS, in a non-randomized comparison. We noticed a numerical advantage for the pembrolizumab combinations vs. the dual nivolumab and ipilimumab combination strategy (although the advantage was not statistically significant).

The multivariate analysis for OS found an independent unfavorable prognostic role for performance status 2, older age, early use of corticoids, and an elevated (>3.81) neutrophil-to-lymphocyte ratio.

Long-term follow-up is only available at the present time for the dual immunotherapy cohorts (7 years for cohort 1A, nivolumab + ipilimumab, and 5 years for cohort 1B, nivolumab + ipilimumab + short course chemotherapy). We can confirm a proportion of long-term survivors in line with those from the registration studies (30.5% at 4 and 5 years in cohort 1A; 25% and 18.8% at 4 and 5 years in cohort 1B), thus opening a window of hope for metastatic NSCLC patients.

## Figures and Tables

**Table 1:** Patient characteristics at baseline (n = 122), overall, and in each of the three treatment cohorts.

Characteristic	All Patients * (n = 122) n (%)	Cohort 1A (n = 18) n (%)	Cohort 1B (n = 33) n (%)	Cohort 2 (n = 71) n (%)	*p*
Age (years), median (range)	62 (41–82)	62.5 (48–75)	62 (44–76)	62 (41–82)	0.91
≤65	83 (68)	13 (72.2)	22 (66.7)	48 (67.6)	
>65	39 (32)	5 (27.8)	11 (33.3)	23 (32.4)	
Gender					0.94
Male	90 (73.8)	14 (77.8)	23 (69.7)	53 (74.6)	
Female	32 (26.2)	12 (22.2)	10 (30.3)	18 (25.4)	
ECOG PS					0.7
0	5 (4.1)	-	2 (6.1)	3 (4.2)	
1	109 (89.3)	18 (100)	31 (93.9)	60 (84.5)	
2	8 (6.6)	-	-	8 (11.3)	
BMI, median (range)	24.9 (14.4–36.5)	24.9 (18.1–34.5)	24.2 (18.2–35.3)	25.5 (14.3–36.4)	0.97
≤18.5	6 (4.9)	1 (5.6)	1 (3)	4 (5.6)	
18.5–24.9	57 (46.7)	9 (50)	18 (54.5)	30 (42.3)	
25–29.9	35 (28.7)	5 (27.8)	7 (21.2)	23 (32.4)	
≥30	24 (19.7)	3 (16.7)	7 (21.2)	14 (19.7)	
Smoking status					0.97
Never smoker	17 (13.9)	3 (16.7)	6 (18.2)	8 (11.3)	
Active smoker	27 (22.1)	1 (5.6)	5 (15.2)	21 (29.6)	
Ex-smoker	78 (64)	14 (77.8)	22 (66.7)	42 (59.2)	
Histology					0.72
Non-squamous adenocarcinoma	82 (67.2)	9 (50)	23 (69.7)	50 (70.4)	
Non-squamous other	1 (0.8)	-	1 (3)	-	
Non-squamous large cell	3 (2.5)	-	1 (3)	2 (2.8)	
Squamous	36 (29.5)	9 (50)	8 (24.2)	19 (26.8)	
Stage (AJCC 8)					0.29
IVA	54 (44.3)	11 (61.1)	14 (42.4)	29 (40.8)	
IVB	68 (55.7)	7 (38.9)	19 (57.6)	42 (59.2)	
Metastatic site					0.81
Lung	77 (63.1)	10 (55.6)	23 (69.7)	44 (62)	
Pleural	37 (30.3)	8 (44.4)	15 (45.5)	14 (19.7)	
Bone	32 (26.2)	3 (16.7)	10 (30.3)	19 (26.8)	
CNS (pretreated, asymptomatic)	27 (22.1)	5 (27.8)	7 (21.2)	15 (21.1)	
Liver	26 (21.3)	3 (16.7)	10 (30.3)	13 (18.3)	
Adrenal	24 (19.7)	2 (11.1)	8 (24.2)	14 (19.7)	
Other	24 (19.7)	1 (5.6)	5 (15.2)	18 (25.4)	
Number of metastatic sites					0.04
1–2	87 (71.3)	15 (83.3)	18 (54.5)	54 (76.1)	
≥3	35 (28.7)	3 (16.7)	15 (45.5)	17 (23.9)	
PD-L1					<0.01
Not evaluated	67 (54.9)	-	30 (90.9)	37 (52.1)	
<1%	26 (21.3)	6 (33.3)	-	20 (28.2)	
≥1%	13 (10.7)	12 (66.7)	-	1 (1.4)	
≥50%	6 (4.9)	-	1 (3)	5 (7)	
1–49%	10 (8.2)	-	2 (6.1)	8 (11.3)	
Actionable mutations **					
Yes	8 (6.6)				
KRAS G12C	3 (2.5)				
cMET amplification	2 (1.6)				
RET	1 (0.8)				
ALK (rebiopsy at progression)	1 (0.8)				
EGFR (rebiopsy at progression)	1 (0.8)				
No	114 (93.4)				
Hemoglobin (g/dL), median (range)	12.9 (8.4–16.1)	12.8 (9.8–15.1)	13.4 (8.5–16.1)	12.9 (8.4–15.8)	0.64
≤13	67 (54.9)	11 (61.1)	16 (48.5)	40 (56.3)	
>13	55 (45.1)	7 (38.9)	17 (51.5)	31 (43.7)	
Neutrophils (×10^3^/µL), median (range)	7.05 (1.9–67.1)	6.6 (3.6–67.1)	6.9 (2.1–26.4)	7.2 (1.9–27.8)	0.56
1.8–6.98	60 (49.2)	10 (55.6)	18 (54.5)	32 (45.1)	
≥6.99	62 (50.8)	8 (44.4)	15 (45.5)	39 (54.9)	
Lymphocytes (×10^3^/µL), median (range)	1.6 (0.5–6.9)	1.9 (0.9–3.0)	1.7 (0.5–6.9)	1.4 (0.5–4.4)	0.03
≤1.5	53 (43.4)	5 (27.8)	10 (30.3)	38 (53.5)	
>1.5	69 (56.6)	13 (72.2)	23 (69.7)	33 (46.5)	
Neut./Lymph. ratio, median (range)	4.3 (0.9–22.3)	3.7 (1.5–22.2)	4.0 (0.9–18.2)	4.8 (1.3–18.2)	0.32
≤3.81	48 (39.3)	9 (50)	15 (45.5)	24 (33.8)	
>3.81	74 (60.7)	9 (50)	18 (54.5)	47 (66.2)	
Platelets (×10^3^/µL), median (range)	316.5 (127–875)	279 (158–586)	326 (127–875)	317 (147–722)	0.96
≤450	102 (83.6)	15 (83.3)	28 (84.8)	59 (83.1)	
>450	20 (16.4)	3 (16.7)	5 (15.2)	12 (16.9)	
LDH (U/L), median (range)	231 (130–1523)	209 (140–1523)	231 (130–1523)	225 (130–799)	0.1
≤225	42 (34.4)	11(61.1)	11(33.3)	20(28.2)	
>225	46 (37.7)	7(38.9)	22(66.7)	17(23.9)	
Not determined	34 (27.9)	-	-	34(47.9)	
Corticoids in the first month					0.54
Yes	31 (25.4)	7 (38.9)	7 (21.2)	17 (23.9)	
No	91 (74.6)	11 (61.1)	26 (78.8)	54 (76.1)	
Previous palliative radiotherapy					0.94
Yes	33 (27)	5 (27.8)	10 (30.3)	18 (25.4)	
No	89 (73)	13 (72.2)	23 (69.7)	53 (74.6)	
Treatment group					
Cohort 1A (CheckMate-227 protocol)	18 (14.8)				
Cohort 1B (CheckMate-9LA protocol)	33 (27)				
Non-squamous	25 (20.5)				
Squamous	8 (6.5)				
Cohort 2	71 (58.2)				
Non-squamous, KeyNote-189 protocol	52 (42.6)				
Squamous, KeyNote-407 protocol	19 (15.6)				

* All patients column represents the simple sum of cohorts 1A, 1B, and 2 for descriptive analysis; patients were successively enrolled in the three cohorts as described in text, and no randomization was performed. ** due to low numbers, individual cohort distribution was not calculated.

**Table 2:** Patient follow-up.

Treatment Protocol	n	Median Follow-Up, Range (Months)
Cohort 1A (CM-227) (Jan 2016–Dec 2017)	18	83 (77.8–84.4)
Cohort 1B (CM-9LA) (Jan 2018–Jul 2019)	33	59 (49.1–63.1)
Cohort 2 (KN-189 and KN-407) Aug 2019–Jun 2023	71	14.2 (2.9–48.3)
Total	122	20 (2.9–84.4)

**Table 3:** Univariate and multivariate analysis of prognostic factors for overall and progression-free survival.

			Progression-Free Survival						Overall Survival					
Category	Prognostic Factor	n	Median Survival (Mo.)	2-Year Survival Rate (%)	95% CI (%)	Univariate Analysis, *p*	Multivariate Analysis		Median Survival (Mo.)	2-year Survival Rate (%)	95% CI (%)	Univariate Analysis, *p*	Multivariate Analysis	
							HR (95%CI)	*p*					HR (95%CI)	*p*
Cohort	1A (CheckMate 227)	18	10.1	32	15.4–55.8	0.71			24.2	55%	33–75.1	0.25		
	1B (CheckMate 9LA)	33	8.4	34	20.5–51.8				13.7	34%	20.4–51.7			
	2 (KeyNote 189/407)	71	12.7	35	23.4–48.1				24.2	53%	39.3–66.9			
Treatment group	Nivolumab + Ipilimumab	51	8.6	34	22.3–47.8	0.41			14	42%	29.2–55.6	0.18		
	Pembrolizumab	71	12.7	35	23.4–48.1				>24	53%	39.3–66.9			
1st line objective response	PD	12	1	0	0–0	<0.01			2.4	0%	0–0	<0.01		
	CR + PR	51	>24	64	49.6–77.0				>24	77%	62.5–87.1			
	SD	59	8	16	8–28.8				13.9	31%	18.9–46.3			
Age	≤60	48	16.6	42	28.9–57.3	0.12			>24	60%	45–74	0.04	1.03 (1.00–1.07)	0.02
	>60	74	9.9	30	20–42.2				14.9	41%	29.2–53.7			
Gender	Female	32	16.6	47	30.6–64.7	0.29			>24	67%	49.1–81	0.29		
	Male	90	10.7	31	21.4–41.7				20.3	43%	31.9–54.2			
ECOG PS	2	8	3.5	0	0–0	<0.01	2.04 (1.09–3.81)	0.02	8.2	0%	0–0	<0.01	2.17 (1.08–4.36)	0.02
	0–1	114	12.6	38	28.9–47.7				>24	52%	28.8–47.6			
BMI	<18.5	6	5.1	17	3–56.4	0.14			7.4	21%	3.8–63.6	0.05	1.00 (0.95–1.06)	0.75
	18.5–24.9	57	11.5	31	19.9–45.3				21.2	49%	33.3–61.2			
	25.0–29.9	35	12	37	22.4–54.3				21.9	49%	32–66.2			
	30+	24	14.8	48	28.8–67.4				>24	60%	39.1–77.3			
Smoking status	Former smoker	78	10.5	32	22.4–44.4	0.13			21.5	48%	36.1–60.1	0.23		
	Active smoker	27	21.7	49	30.7–67.3				>24	61%	40–77.8			
	Never smoker	17	8.4	26	10.1–51.6				13.6	34%	14.5–61.5			
Histology	Non-squamous	86	12.5	40	30.1–51.7	0.34			>24	55%	44.1–66.2	0.45		
	Squamous	36	9.9	22	11–39.6				17.4	33%	18.5–52.1			
AJCC stage	IVA	54	8.4	48	34.4–62.4	<0.01	1.35 (0.77–2.38)	0.28	>24	57%	42.4–70.5	0.04	1.43 (0.74–2.76)	0.28
	IVB	68	25.5	25	15.7–37				13.8	42%	29.9–55.4			
Steroid use in the first month	No	91	13.3	39	28.4–49.8	0.02	1.44 (0.87–2.39)	0.15	10.5	38%	22.6–56.4	0.02	1.79 (1.01–3.16)	0.04
	Yes	31	4.1	24	12.1–41.5				>24	52%	40.4–63.3			
Palliative radiotherapy	No	89	14.8	41	30.8–52.2	0.02			24.1	52%	40.2–62.6	0.14		
	Yes	33	8	18	8.1–35.5				13.9	41%	24.4–59.8			
Number of metastatic sites	0–2	87	17	42	31.6–54	<0.01			>24	59%	46.9–69.5	<0.01		
	3+	35	7.2	17	8.1–32.7				12.1	26%	13.3–43.6			
Bone metastases	No	90	14.7	42	31.6–53.1	<0.01	1.67 (0.99–2.81)	0.05	>24	55%	43–65.6	<0.01	1.64 (0.91–2.95)	0.09
	Yes	32	6.7	15	6.1–33.7				11.4	32%	17.6–51.3			
Liver metastases	No	96	13.3	38	28.6–49	0.03	1.25 (0.70–2.25)	0.44	12.2	33%	16.8–54.2	0.05	1.23 (0.63–2.4)	0.53
	Yes	26	7.2	23	10.6–42.9				24.2	53%	41.6–63.3			
CNS metastasis	No	95	12.2	36	26.7–47.2	0.42			>24	52%	32.9–70.2	0.73		
	Yes	27	10.5	30	15.6–49.5				21.2	48%	36.8–58.9			
Adrenal metastasis	No	98	12.7	36	26.3–46.3	0.35			24.2	51%	40.4–61.9	0.2		
	Yes	24	7.4	32	16–52.8				13	38%	20.5–59.7			
Pleural metastasis	No	85	11	37	26.5–48	0.87			24.1	52%	39.7–63.4	0.6		
	Yes	37	12.5	33	20.1–50				18.4	43%	28.3–59.8			
Lung metastasis	No	45	5.2	25	14.1–40.4	0.08			13.3	43%	28.5–58.7	0.08		
	Yes	77	12.6	41	29.8–52.7				24.1	52%	39.5–63.9			
Other metastasis	No	98	12.5	39	29.2–49.5	0.17			14.5	30%	12–56.8	0.95		
	Yes	24	8.7	18	6.9–40.3				24.2	52%	41.2–62.1			
Hemoglobin (g/dL)	≤13	67	8.7	25	15.5–37.8	0.04	0.64 (0.40–1.05)	0.08	14	41%	28.9–53.7	0.03	0.70 (0.41–1.19)	0.19
	>13	55	17	47	33.6–60.6				>24	59%	43.8–72.2			
LDH (U/L)	≤225	42	8.7	36	22.6–52.6	0.24			>24	50%	34.5–65.9	0.29		
	>225	46	8.4	26	15.2–40.6				13.9	36%	22.6–52.4			
Platelets (×1000/µL)	≤450	102	11.3	33	23.8–43.1	0.22			21.9	48%	37.1–58.6	0.92		
	>450	20	19.4	46	26.1–68.1				>24	52%	30.5–72.3			
Neutrophils (×1000/µL)	1.8–6.98	60	10.9	39	26.5–52.1	0.6			>24	55%	40.7–68.1	0.08		
	6.99+	62	12	32	21.1–45.2				20.3	43%	30.5–56.6			
Lymphocytes (×1000/µL)	≤1.5	53	10.4	41	15.4–40.7	0.33			14.9	40%	26.8–55.6	0.25		
	>1.5	69	12.6	26	29.9–53.8				>24	55%	42.4–67.1			
Neutrophil-to-lymphocyte ratio	≤3.81	48	16.6	45	31.1–59.5	0.05	1.23 (0.76–1.99)	0.39	>24	63%	47.1–75.8	<0.01	1.81 (1.04–3.15)	0.03
	3.81+	74	10.2	29	18.8–40.7				14.9	39%	27.6–52.1			
PDL1 status	Undetermined	67	9.9	29	19.3–42.1	0.23			18	40%	27.6–53.8	0.36		
	Negative	26	12.2	48	30.2–67.2				>24	62%	41.4–78.7			
	Positive	29	16.6	36	19.9–56.1				>24	55%	36.3–72.5			
Actionable mutations	Yes	8	11.5	12	2.2–47.1	0.26			17.4	50%	21.5–78.4	0.44		
	No	114	11	37	28.4–47				21.9	48%	38.1–58.4			

**Table 4:** Univariate analysis of prognostic factors for objective response and clinical benefit.

				Objective Response		Clinical Benefit	
Category	Prognostic Factor	PD n (%)	SD n (%)	CR + PR n (%)	*p*	CR + PR + SD n (%)	*p*
Age	≤65	7 (8.4%)	37 (44.6%)	39 (47%)	0.36	76 (91.6%)	0.67
	>65	5 (12.8%)	22 (56.4%)	12 (30.8%)		34 (87.2%)	
	≤61 *	2 (3.7%)	25 (46.3%)	27 (50%)	0.07	52 (96.3%)	0.04
	>61	10 (14.7%)	34 (50%)	24 (35.3%)		58 (85.3%)	
Gender	Female	3 (9.4%)	12 (37.5%)	17 (53.1%)	0.4	29 (90.6%)	0.81
	Male	9 (10%)	47 (52.2%)	34 (37.8%)		81 (90%)	
ECOG PS	0		3 (60%)	2 (40%)	0.03	5 (100%)	<0.01
	1	8 (7.3%)	53 (48.6%)	48 (44%)		101 (92.7%)	
	2	4 (50%)	3 (37.5%)	1 (12.5%)		4 (50%)	
BMI	<18.5	2 (33.3%)	4 (66.7%)		0.72	4 (66.7%)	0.61
	18.5–24.9	4 (7%)	25 (43.9%)	28 (49.1%)		53 (93%)	
	25.0–29.9	4 (11.4%)	19 (54.3%)	12 (34.3%)		31 (88.6%)	
	≥30	2 (8.3%)	11 (45.8%)	11 (45.8%)		22 (91.7%)	
Histology	Non-squamous	6 (7%)	41 (47.7%)	39 (45.3%)	0.34	80 (93%)	0.19
	Squamous	6 (16.7%)	18 (50%)	12 (33.3%)		30 (83.3%)	
AJCC stage	IVA	5 (9.3%)	21 (38.9%)	28 (51.9%)	0.12	49 (90.7%)	0.85
	IVB	7 (10.3%)	38 (55.9%)	23 (33.8%)		61 (89.7%)	
Metastasis site							
Lung	Yes	4 (5.2%)	38 (49.4%)	35 (45.5%)	0.15	73 (94.8%)	0.05
	No	8 (17.8%)	21 (46.7%)	16 (35.6%)		37 (82.2%)	
Pleural	Yes	6 (16.2%)	16 (43.2%)	15 (40.5%)	0.47	31 (83.8%)	0.22
	No	6 (7.1%)	43 (50.6%)	36 (42.4%)		79 (92.9%)	
Bone	Yes	4 (12.5%)	19 (59.4%)	9 (28.1%)	0.3	28 (87.5%)	0.81
	No	8 (8.9%)	40 (44.4%)	42 (46.7%)		82 (91.1%)	
CNS	Yes	2 (7.4%)	16 (59.3%)	9 (33.3%)	0.62	25 (92.6%)	0.91
	No	10 (10.5%)	43 (45.3%)	42 (44.2%)		85 (89.5%)	
Liver	Yes	4 (15.4%)	15 (57.7%)	7 (26.9%)	0.34	22 (84.6%)	0.48
	No	8 (8.3%)	44 (45.8%)	44 (45.8%)		88 (91.7%)	
Adrenal	Yes	2 (8.3%)	12 (50%)	10 (41.7%)	0.98	22 (91.7%)	0.92
	No	10 (10.2%)	47 (48%)	41 (41.8%)		88 (89.8%)	
Other	Yes	1 (4.2%)	13 (54.2%)	10 (41.7%)	0.78	23 (95.8%)	0.51
	No	11 (11.2%)	46 (46.9%)	41 (41.8%)		87 (88.8%)	
Number of metastatic sites	0–2	9 (10.3%)	36 (41.4%)	42 (48.3%)	0.08	78 (89.7%)	0.97
	≥3	3 (8.6%)	23 (65.7%)	9 (25.7%)		32 (91.4%)	
PDL1 status	Undetermined	5 (7.5%)	39 (58.2%)	23 (34.3%)	0.26	62 (92.5%)	0.14
	Negative (<1%)	1 (3.8%)	12 (46.2%)	13 (50%)		25 (96.2%)	
	Positive (≥1%)	6 (20.7%)	8 (27.6%)	15 (51.7%)		23 (79.3%)	
Hemoglobin (g/dL)	≤13	10 (14.9%)	32 (47.8%)	25 (37.3%)	0.1	57 (85.1%)	0.04
	>13	2 (3.6%)	27 (49.1%)	26 (47.3%)		53 (96.4%)	
Neutrophils (×1000/µL)	1.8–6.98	5 (8.3%)	31 (51.7%)	24 (40%)	0.11	55 (91.7%)	0.58
	6.99+	7 (11.3%)	28 (45.2%)	27 (43.5%)		55 (88.7%)	
Lymphocytes (×1000/µL)	≤1.5	7 (13.2%)	30 (56.6%)	16 (30.2%)	0.07	46 (86.8%)	0.27
	>1.5	5 (7.2%)	29 (42%)	35 (50.7%)		64 (92.8%)	
Neutrophil-to-lymphocyte ratio	≤3.81	1 (2.1%)	27 (56.2%)	20 (41.7%)	0.11	47 (97.9%)	0.04
	3.81+	11 (14.9%)	32 (43.2%)	31 (41.9%)		63 (85.1%)	
Platelets (×1000/µL)	≤450	9 (8.8%)	51 (50%)	42 (41.2%)	0.84	93 (91.2%)	0.66
	>450	3 (15%)	8 (40%)	9 (45%)		17 (85%)	
LDH (U/L)	≤225	3 (7.1%)	25 (59.5%)	14 (33.3%)	0.71	39 (92.9%)	0.39
	>225	7 (15.2%)	25 (54.3%)	14 (30.4%)		39 (84.8%)	
	Undetermined	2 (5.9%)	9 (26.5%)	23 (67.6%)		32 (94.1%)	
Steroid use in the first month	Yes	7 (22.6%)	16 (51.6%)	8 (25.8%)	0.03	24 (77.4%)	0.02
	No	5 (5.5%)	43 (47.3%)	43 (47.3%)		86 (94.5%)	
Treatment group	Nivolumab + Ipilimumab	7 (13.7%)	27 (52.9%)	17 (33.3%)	0.2	44 (86.3%)	0.22
	Pembrolizumab	5 (7%)	32 (45.1%)	34 (47.9%)		66 (93%)	
Cohort	1A CheckMate-227	4 (22.2%)	7 (38.9%)	7 (38.9%)	0.59	14 (77.8%)	0.53
	1B (non-Sq) CheckMate-9LA	1 (4%)	15 (60%)	9 (36%)		24 (96%)	
	1B (Sq) CheckMate-9LA	2 (25%)	5 (62.5%)	1 (12.5%)		6 (75%)	
	2 (non-Sq) KeyNote-189	4 (7.7%)	21 (40.4%)	27 (51.9%)		48 (92.3%)	
	2 (Sq) KeyNote-407	1 (5.3%)	11 (57.9%)	7 (36.8%)		18 (94.7%)	

* Statistical significance was reached for this age threshold.

## Data Availability

The raw data supporting the conclusions of this article will be made available by the authors on request.
